# Antiviral Potential of Azelastine against Major Respiratory Viruses

**DOI:** 10.3390/v15122300

**Published:** 2023-11-23

**Authors:** Katrin Fischhuber, Zoltán Bánki, Janine Kimpel, Natalie Kragl, Annika Rössler, Annika Bolze, Brigitte Muellauer, Joachim Angerer, Gábor Nagy, Eszter Nagy, Valeria Szijarto

**Affiliations:** 1 CEBINA GmbH, 1030 Vienna, Austria; 2 Institute of Virology, Medical University of Innsbruck, 6020 Innsbruck, Austria; zoltan.banki@i-med.ac.at (Z.B.); annika.bolze@student.i-med.ac.at (A.B.);

**Keywords:** antiviral, drug repurposing, respiratory viruses, RSV, azelastine

## Abstract

The Coronavirus Disease 2019 (COVID-19) pandemic and the subsequent increase in respiratory viral infections highlight the need for broad-spectrum antivirals to enable a quick and efficient reaction to current and emerging viral outbreaks. We previously demonstrated that the antihistamine azelastine hydrochloride (azelastine-HCl) exhibited in vitro antiviral activity against SARS-CoV-2. Furthermore, in a phase 2 clinical study, a commercial azelastine-containing nasal spray significantly reduced the viral load in SARS-CoV-2-infected individuals. Here, we evaluate the efficacy of azelastine-HCl against additional human coronaviruses, including the SARS-CoV-2 omicron variant and a seasonal human coronavirus, 229E, through in vitro infection assays, with azelastine showing a comparable potency against both. Furthermore, we determined that azelastine-HCl also inhibits the replication of Respiratory syncytial virus A (RSV A) in both prophylactic and therapeutic settings. In a human 3D nasal tissue model (MucilAir^TM^-Pool, Epithelix), azelastine-HCl protected tissue integrity and function from the effects of infection with influenza A H1N1 and resulted in a reduced viral load soon after infection. Our results suggest that azelastine-HCl has a broad antiviral effect and can be considered a safe option against the most common respiratory viruses to prevent or treat such infections locally in the form of a nasal spray that is commonly available globally.

## 1. Introduction

Viral airway infections are among the most common human infectious diseases. While upper respiratory tract infections are usually mild and self-limiting, lower respiratory tract infections are often severe, difficult to treat, and cause high mortality, especially in infants and the elderly [1,2]. Common respiratory viruses have adapted to human-to-human transmission and have become endemic. Others (like influenza viruses) circulate on a global scale and cause seasonal epidemics. Viruses typically have a fine-tuned evolution that allows the evasion of the pre-existing immunity acquired through preceding infections while retaining virulence and transmission potential. This leads to endemicity, i.e., the virus’s presence in a pre-exposed population for a longer period of time. The exchange of genetic material with similar animal viruses, however, may cause the quantum leap evolution of immune evasion and/or virulence, and such emerging ‘hybrid’ viruses can become epidemic or even pandemic [3]. The emergence of such pandemic strains is difficult to predict and, therefore, poses a huge public health risk [4].

The treatment options for the infections caused by the numerous respiratory viruses are limited and are often restricted to supportive therapy. In the case of upper respiratory viruses, locally acting antivirals are expected to limit viral propagation and dissemination to the lower airways, which could cause chronic or systemic infections. During the COVID-19 pandemic, several nasal sprays/applications containing antiviral compounds or even vaccine antigens were investigated [5]. 

Vaccines are available only against pathogens causing the most severe infections. Moreover, these vaccines are suboptimal in several aspects, including a short duration of immunity, low efficacy in immunocompromised populations, and a narrow spectrum of cross-protection for the various subtypes of a given pathogen. Furthermore, vaccine-based (and infection-induced) immunity causes evolutionary pressure that selects variants that are capable of evading the immunity gained upon vaccination [6]. This is best represented by the annual need for new seasonal flu vaccines and the even more rapid evasion of vaccine immunity against SARS-CoV-2 variants during the recent COVID-19 pandemic [7]. The limitation of vaccines, together with the huge number of potential viral pathogens (against most of which no vaccines are available) necessitates the development of broadly acting, non-specific prophylactic measures against respiratory viruses. In contrast to specific antivirals that target viral enzymes, broad-spectrum antivirals must interfere with general host cellular machinery or the compartments that are utilized by various unrelated viral pathogens. Safety considerations, however, set limits on this approach. The repurposing of licensed drugs allows an assessment of antiviral efficacy without safety concerns, which can facilitate the development of broad antiviral drugs [8]. Broad-spectrum antiviral agents (BSAA) are defined in the literature as agents acting against different viruses within the same family or from different viral families [9]. More specifically, the term BSAA is used for compounds acting on viruses from at least two viral families [10].

It was previously shown, using in silico methods, that the approved drug azelastine-HCl has antiviral activity. Azelastine-HCL inhibited SARS-CoV-2 replication in in vitro assays, and a clinical study with a commercially available nasal spray containing azelastine-HCl demonstrated a reduction in the viral load of SARS-CoV-2 in the nasopharynx [11]. Here, we provide in vitro evidence for the antiviral activity of azelastine-HCl against unrelated common respiratory viruses. Upon confirming its broad-spectrum efficacy in future clinical studies, azelastine-HCl nasal sprays may represent a useful prophylactic tool that can prevent or ameliorate upper respiratory viral infections and limit the spread of viral pathogens, especially pandemic ones [12]. 

## 2. Materials and Methods

### 2.1. Chemicals

Azelastine hydrochloride (azelastine-HCl) was purchased from a commercial source (Sigma-Aldrich (St. Louis, MO, USA), A7611) and dissolved in DMSO. Alternatively, the commercially available azelastine-HCl nasal spray Pollival^®^ (URSAPHARM Arzneimittel GmbH, Saarbruecken, Germany) was used. The buffer of Pollival^®^ was provided by the manufacturer (URSAPHARM Arzneimittel GmbH, Saarbruecken, Germany) and was identical to the placebo used in clinical trials by the company [11].

### 2.2. Viruses, Primers and Cells

The SARS-CoV-2 omicron variant (B.1.1.529, BA.1 subtype ID: EPI_ISL_6902053) was isolated at the Medical University of Innsbruck. The human coronavirus 229E (HCoV-229E) was derived from the National Collection of Pathogenic Viruses (NCVP). The RSV Long strain (kindly provided by T. Grunwald, Fraunhofer Institute for Cell Therapy and Immunology, Leipzig, Germany) was generated via the infection of HEp-2 cells at a low MOI, as described previously [13]. Influenza A H1N1 was isolated from a clinical specimen, A/Switzerland/7717739/2013 (H1N1), as described in [14]. 

Vero cells stably overexpressing the human serine protease TMPRSS2 and ACE2 receptor were generated as described elsewhere [15] and cultured in DMEM (Merck, Darmstadt, Germany) containing 2% FBS (PAN-Biotech, Aidenbach, Germany). 

MRC-5 cells were purchased from ECACC at passage number 25 (Cat. No. 05081101) and were grown under aseptic standard cell culture conditions (5% CO_2_, 37 °C) in DMEM (high glucose, GlutaMAX™ Supplement; Gibco, Thermo Fisher Scientific; Waltham, MA, USA) supplemented with 10% heat-inactivated (HI) FBS (Sigma Aldrich, St. Louis, MO, USA) and 1% penicillin–streptomycin (10,000 units penicillin and 10 mg streptomycin, Sigma Aldrich).

HEp-2 cells (ATCC/CCL-23^TM^) used for RSV assays were cultivated in DMEM (Sigma Aldrich) supplemented with 2 mM of L-glutamine (Gibco, Thermo Fischer Scientific, Vienna, Austria) and 10% FCS (Gibco, Thermo Fischer Scientific). 

The human nasal tissue MucilAir^TM^ (Epithelix Sarl, Geneve, Switzerland) batch number Pool9 Oxy 11 was derived from a pool of nasal epithelium from 14 donors (aged 32–58 years) and was cultured at the air–liquid interface to promote the differentiation and polarization to fully ciliated epithelia [16].

The primers used for virus detection are listed in Table 1.

### 2.3. Infection of the Vero-TMPRSS2/ACE2 Cell Line by SARS-CoV-2 Omicron

The in vitro testing of azelastine-HCl against the omicron variant was performed as described [18], including the quantification of the viral genome, except for the modification that the cells were infected with the virus for a prolonged time (2 h). 

### 2.4. Infection Model with 229E

MRC-5 cells were seeded on two 96-well plates at a density of 2.5 × 10^4^/well the day before the experiment. Prior to infection, the supernatant was removed and replaced with an equal volume (50 µL) of the medium with the HCo-V-229E virus (MOI of 0.001) or without any virus (control cells) and the 2–12 µM azelastine-HCl or a respective DMSO dilution (vehicle control). After 60 min of incubation at 5% CO_2_ at 33 °C, the supernatant was replaced with fresh medium containing the same concentration of azelastine-HCl or DMSO. The cells were incubated at 5% CO_2_ at 33 °C for 72 h post-infection (p.i.). All samples were run in quadruplicates. The antiviral effect was assessed via viral genome copy determination from the supernatant for 72 h p.i. using RT-qPCR. For this, the RNA was isolated with a Monarch^TM^ Total RNA Miniprep Kit (New England Biolabs Inc., Ipswich, MA, USA) and N gene-specific primers at a 0.5 µM final concentration and using the Luna^®^ Universal One-Step RT-qPCR Kit (New England Biolabs Inc.) following the manufacturer’s recommendations. RNA copy numbers were calculated with the help of standard curves generated from genomic RNA from Quantitative Genomic RNA from HCoV-229E purchased from American Type Culture Collection (ATCC, Virginia, USA) via LGC (Wesel, Germany).

### 2.5. In Vitro Testing against RSV

HEp-2 cells were plated at a density of 1.75 × 10^4^ cells/well in 100 μL of DMEM supplemented with 2 mM L-glutamine/10% FCS in a 96-well cell culture microplate and were cultured overnight at 37 °C. The next day, HEp-2 cells were infected with RSV at a predetermined dilution, resulting in approximately 300 infected cells when stained for 24 h post-infection, as described below. HEp-2 cells were infected with RSV in the absence or presence of different concentrations of azelastine-HCl ranging from 50 μM to 0.024 μM. DMSO was used as a vehicle control at corresponding concentrations. After 24 h of infection, RSV-infected cells were detected with immunofluorescence staining. For this, the supernatant was removed, and the cells were fixed with 95% ethanol for 5 min. After washing with Tris-buffered saline (TBS)/0.1% BSA, the cells were incubated with an RSV-specific recombinant monoclonal antibody, Palivizumab (Synagis, AstraZeneca, Vienna, Austria) at a concentration of 20 μg/mL for 1 h at room temperature. After washing, the cells were stained with an Alexa Fluor^TM^ Plus 488-labeled polyclonal goat anti-human IgG (H + L) (Invitrogen^TM^, Thermo Fischer Scientific, Vienna, Austria) at a concentration of 4 μg/mL for 1 h at room temperature. Plates were washed 4 times, residual liquid was carefully removed, and green fluorescent spots were counted using an ImmunoSpot^®^ analyzer (C.T.L. Europe, Rutesheim, Germany). The percentage of inhibition of RSV infection was calculated relative to 100% infection, counted in HEp-2 cells with RSV only. 

### 2.6. Influenza Induced Deregulation Model Using the Human Nasal 3D Tissue MucilAir^TM^

This study was run at the independent research facility Epithelix Sarl (Geneve, Switzerland).

The nasal epithelium was cultured according to the manufacturer’s instructions. Pollival^®^ was diluted with its own buffer at 1:5 or 1:10 and applied to the apical surface of the cells 10 min before infection with 10^5^ H1N1 viral particles in 100 µL of the culture medium on the apical side. After 3 h of incubation at 34 °C, 5% CO_2_, with 100% humidity, the apical surface of the cells was washed 3 times with culture media to remove the unbound viruses. Residual viruses after washes were collected using a 20 min apical wash and were quantified using qPCR to establish a baseline for viral growth. New viral particles were collected with 20 min apical washes at 24, 48, 72, and 96 h p.i. Exposure to Pollival^®^ was renewed every day by re-applying it to the apical surface. 

As a negative control, uninfected and untreated cells were included (mock), as well as the buffer of Pollival^®^ was tested alone, as described above. The antiviral drug, Oseltamivir was diluted in 0.9% NaCl (1 mM) and used at 10 µM in the basolateral medium concomitantly with viral inoculation and exposure renewed every day. 

To monitor the effect of Pollival^®^ on the MucilAir^TM^-Pool, non-infected tissues were treated the same way with Pollival^®^. 

All conditions were tested with 3 technical replicates. 

### 2.7. Efficacy Read-Out Monitoring in Influenza Model

The effect on virus replication was assessed via virus genome copy number determination from the apical washes at different time points using Taqman RT-PCR [14]. The effect on cilia was monitored using cilia motion (Cilia Beating Frequency, CBF) and through the determination of the area with active cilia beating using a Sony XCD V60 camera connected to an Olympus BS51 microscope and the proprietary software of Epithelix Sarl (Cilia-X). Cytokines (IL-8 and RANTES) were measured from the basolateral media via a commercial ELISA (IL8—BD Biosciences, Allschwil, Switzerland and Human CCL5/RANTES from R & D Systems, Minneapolis, MN, USA). 

### 2.8. Cytotoxicity Assays

The potential cytotoxic effect of azelastine-HCl against MRC-5 cells was tested with Viral ToxGlo^TM^ according to the manufacturer’s instructions (Promega GmbH, Madison, WI, USA); luminescence was measured with a BioTek Synergy HTX multimode plate reader (BioTek, Winooski, VT, USA). The RLUs of the treated wells were normalized to the mean of non-treated wells and then multiplied by 100.

To determine the cytotoxicity of azelastine-HCl and the corresponding concentrations of DMSO in HEp-2 cultures, the MTT assay was performed using the Cell Meter^TM^ Colorimetric MTT Cell Proliferation Kit (ATT Bioquest, Biomol GmbH, Hamburg, Germany) according to the manufacturer’s instructions.

Cell toxicity on MucilAir^TM^ was detected by measuring trans-epithelial electrical resistance (TEER) with an EVOM volt ohmmeter (World Precision Instruments, Hitchin, UK) and through LDH measurement on non-infected, drug-treated tissues with the Cytotoxicity LDH Assay Kit-WST following the manufacturer’s instruction (Dojindo EU GmbH, Munich, Germany).

### 2.9. Data Analysis and Statistical Analysis

The EC_50_ value of azelastine-HCl was determined with nonlinear regression ((inhibitor) vs. a response–variable slope (four parameter)) using GraphPad Prism 9.5.1 (GraphPad Software, Inc., La Jolla, CA, USA). In the influenza in vitro model, differences between three or more groups were tested via one- or two-way ANOVA with Dunnett’s multiple comparison post-tests using GraphPad Prism 6. The differences between the two groups were tested using Student’s *t*-test. Values *p* < 0.05 were considered statistically significant. 

## 3. Results

### 3.1. In Vitro Activity of Azelastine against Coronaviruses 

We previously demonstrated the comparable in vitro activity of azelastine-HCl against major genetic variants of SARS-CoV-2. To evaluate the antiviral effect against coronaviruses in general, we tested azelastine against the B.1.1.529 (BA.1) SARS-CoV-2 variant, as well as the seasonal coronavirus, HCoV-229E, in vitro.

**Figure 1:**
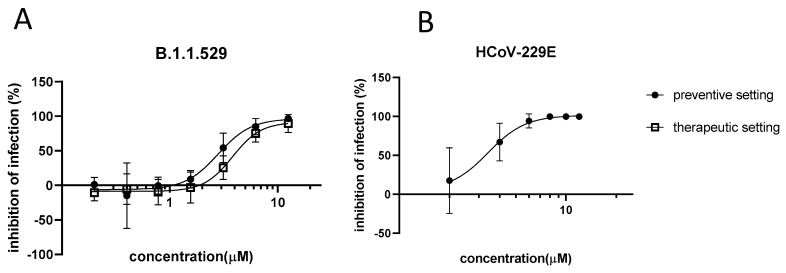
In vitro efficacy of azelastine-HCl against coronaviruses. (**A**) Vero-TMPRSS2/ACE2 cells were infected with the B.1.1.529 variant of SARS-CoV-2 or (**B**) MRC-5 cells were infected with HCoV-229E simultaneously with the addition of (**A**) 0.05–12.5 μM or (**B**) 2–12 µM of azelastine. After 48 h (**A**) or 72 h (**B**) p.i., the viral count was determined using qPCR analysis. Graphs show percent inhibition of infection based on viral genome counts relative to the virus-only control expressed as the mean ± SEM from 3 independent experiments, each with (**A**) 3 or (**B**) 4 technical replicates. The curve was fitted, and EC_50_ was calculated via nonlinear regression using GraphPad Prism.

The anti-SARS-CoV-2 activity of azelastine was tested in a transgenic Vero cell line, overexpressing the human serin protease TMPRSS2 and the ACE2 receptor. We confirmed the retained activity of azelastine-HCl against the BA.1 omicron variant with an EC_50_ of 2.8 µM upon co-administration and 3.8 µM in the therapeutic setting (Figure 1A). These values are in the range of the EC_50_ values previously described against other variants using the same in vitro model (Table S1). 

When infecting MRC-5 cells with the HCoV-229E virus, the administration of azelastine-HCl concomitant to the infection (and re-applied after infection) resulted in a dose-dependent reduction in the viral genome count at 72 h p.i. (Figure 1B). The EC_50_ was similar to that observed against SARS-CoV-2, 3.4 µM.

No drug-related cell toxicity was observed on Vero-TMPRSS2/ACE-2 and MRC-5 cells below 25 µM [18] and 8 µM azelastine-HCl concentrations, respectively.

### 3.2. Prophylactic and Therapeutic Efficacy of Azelastine on RSV Infection of Hep-2 Cells

**Figure 2:**
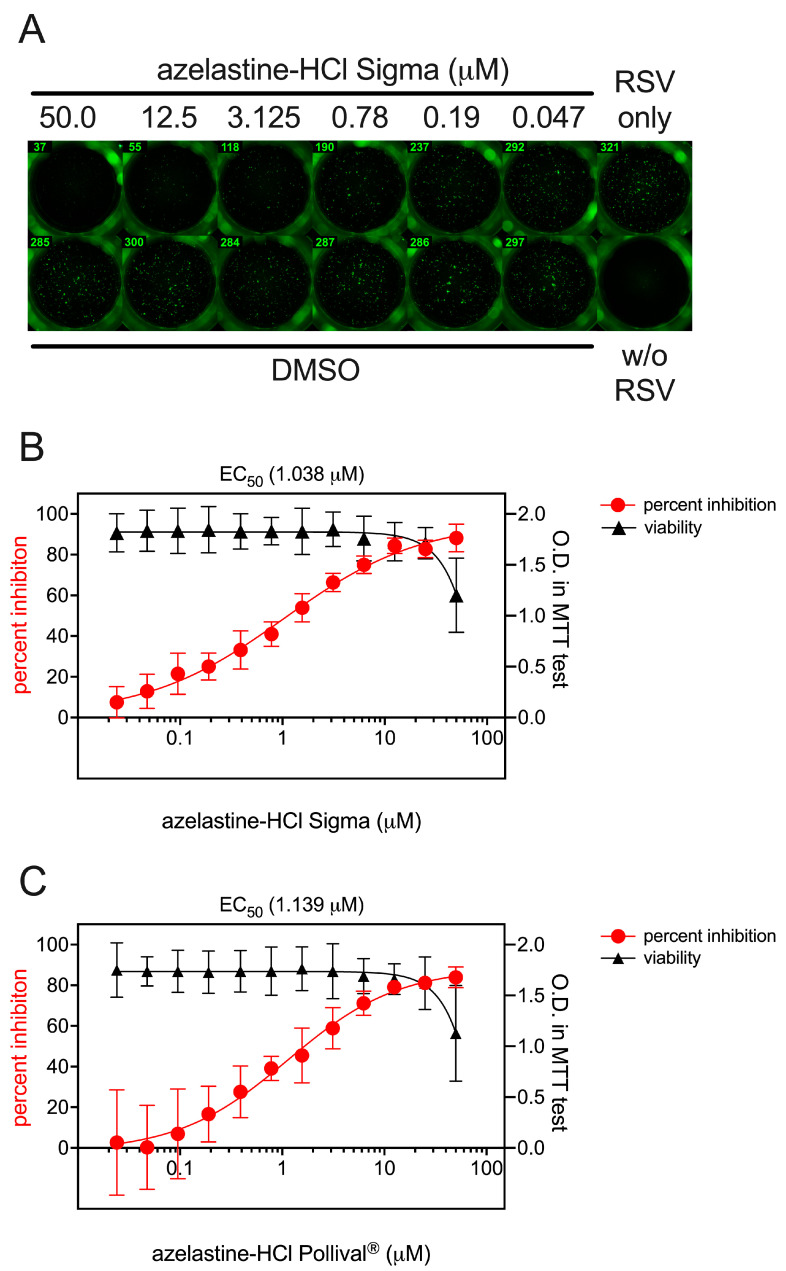
In vitro antiviral effect of azelastine-HCl against RSV. HEp-2 cells were infected with RSV and concomitantly treated with 0.024–50 μM azelastine-HCl. After 24 h, the RSV-infected cells were stained with an RSV-specific recombinant monoclonal antibody followed by Alexa Fluor^TM^ Plus 488-labeled polyclonal goat anti-human IgG staining and the number of RSV-infected cells were counted using an ImmunoSpot^®^ analyzer. (**A**) A representative result of RSV-infected cells treated with azelastine-HCl, DMSO, or non-infected cells (w/o). (**B**) Percent inhibition of RSV infection in the presence of different concentrations of azelastine-HCl as a purified compound or (**C**) azelastine-HCl from the nasal spray Pollival^®^ calculated relative to the infection in virus-only cells (red circles). EC_50_ was calculated via nonlinear regression using GraphPad Prism. The potential cytotoxic effect of azelastine-HCl on the HEp-2 cells was assessed using the MTT assay (black triangles). Graphs show the mean with 95% CI from 8 and 4 independent experiments for RSV inhibition and cell viability, respectively.

HEp-2 cells were infected with RSV, resulting in approximately 300 infected cells per well, as shown by immunofluorescence at 24 h p.i. (Figure 2A, RSV only). HEp-2 cells were also infected in the presence of an azelastine-HCl concentration ranging from 50 μM to 0.024 μM or in the presence of various concentrations of DMSO corresponding to the buffer of respective azelastine-HCl dilutions. While DMSO had no effect on RSV infection in HEp-2 cells, we found a concentration-dependent inhibition of infection by azelastine-HCl (Figure 2A). The EC_50_ for azelastine-HCl against RSV infection was 1.038 μM (Figure 2B). A similar EC_50_ (1.139 μM) was measured when the azelastine-HCl containing nasal spray Pollival^®^ was used (Figure 2C). Importantly, no cytotoxicity was observed in the MTT assay with HEp-2 treated with azelastine-HCl used at a concentration of 25 μM or less (Figure 2B).

To investigate the mode of action of azelastine-HCl on RSV inhibition, we repeated infection experiments using 1 μM of azelastine-HCl. In addition to the previous experimental settings in which RSV and azelastine were applied simultaneously to HEp-2 cells (Supplementary Figure S1, black bars), we performed the experiments in the prophylactic and therapeutic settings by applying azelastine-HCl before or after RSV infections. In the prophylactic setting, HEp-2 cells pre-treated with azelastine-HCl 2 h prior to infection and during infection showed similar infection levels when compared to the application of azelastine-HCl only at the time of infection (pre and co-administration, Supplementary Figure S1). However, in the prophylactic setting, if azelastine-HCl was removed prior to infection, the inhibition of RSV infection was greatly reduced (pre-wash, Supplementary Figure S1). Similarly, the therapeutic setting, where azelastine-HCl was applied 2 h after infection, it resulted in a reduced inhibition of RSV infection (Supplementary Figure S1). Comparable results were obtained when the azelastine-containing nasal spray (Pollival^®^) was used instead of the pure azelastine-HCl compound (Supplementary Figure S1). Taken together, this suggests that the presence of azelastine-HCl during RSV infection is required for optimal inhibition. 

### 3.3. Protective Effect of Azelastine in a Human Nasal 3D Tissue Infection Model of Influenza A

**Figure 3:**
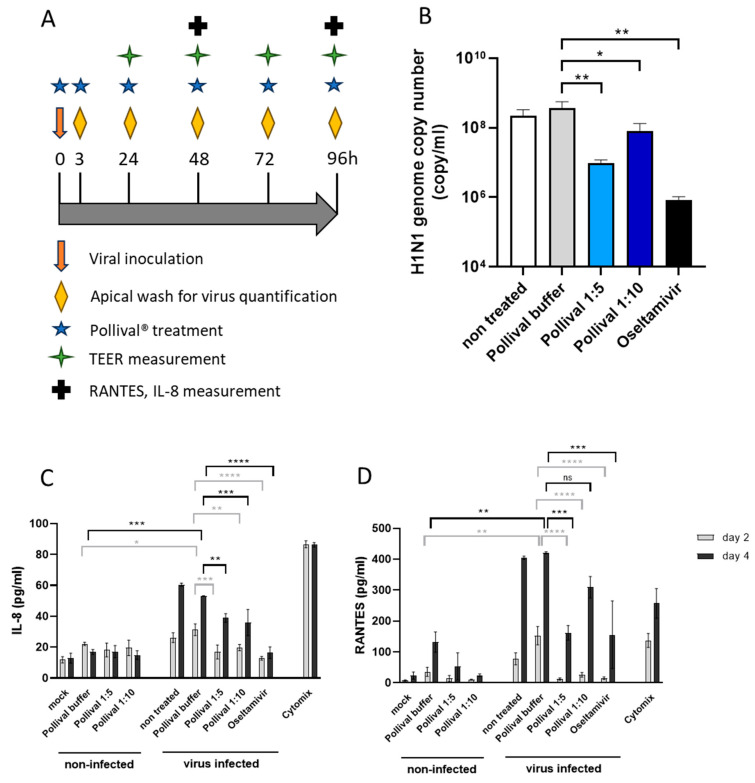
Antiviral effect of azelastine-HCl in an influenza H1N1 infection model on human nasal 3D tissue. (**A**) Experimental scheme. (**B**) H1N1 viral genome count from apical washes at 24 h p.i. measured using a quantitative PCR while the (**C**) IL-8 level and (**D**) RANTES level in basal tissue media after 48 and 96 h p.i. were measured with commercial ELISA kits. Mock indicates non-infected and non-treated tissues. The difference between groups was statistically analyzed with one-way ANOVA (multiple comparisons) using GraphPad Prism, and results were considered significant if *p* < 0.05. * *p* < 0.05; ** *p* < 0.01; *** *p* < 0.001; **** *p* < 0.0001; ns indicates non-significant difference. The graphs show the mean ± SD of the results from 3 tissue samples.

We performed the in vitro testing according to the study scheme shown in Figure 3A. The H1N1 virus showed replication on MucilAir^TM^ with a steady-state plateau (~10^10^ viral genome/mL) that was reached at 48 h p.i. The repeated administration of the 1:5 and 1:10-diluted azelastine-HCl nasal spray significantly reduced the H1N1 genome copy number compared to the vehicle at 24 h (1.57 log10 and 0.65 log10 apical virus reduction, respectively) (Figure 3B); however, the effect was absent after 48 h. The antiviral drug, Oseltamivir, decreased the genome copy number by 2.64–2.66–1.75–0.45 log10 at 24, 48, 72 and 96 h, respectively. 

In addition to the effect on viral replication, the azelastine-HCl nasal spray showed significant tissue protection based on cilia beating. The H1N1 infection decreased CBF in the vehicle-treated culture (0 Hz), whereas exposure to 1:5 diluted Pollival^®^ significantly prevented an H1N1-induced decrease (5.5 Hz) comparable to the effect of Oseltamivir (10.8 Hz) at day 4 (Supplementary Figure S2A). A similar trend, but no statistically significant effect was seen on active cilia area determination, where 1:5 and 1:10-diluted Pollival^®^ increased the active cilia area to 43.7 and 27.7%, respectively, compared to the vehicle (13%) and non-treated cultures (21.7%) (Supplementary Figure S2B). In comparison, Oseltamivir completely prevented the H1N1-induced decrease. 

Importantly, the azelastine-HCl nasal spray significantly reduced the IL-8 and RANTES response of the nasal cultures to the H1N1 infection (Figure 3C,D). IL-8 was comparably reduced by both dilutions of Pollival^®^ to Oselatmivir on day 2 and remained significantly lower compared to the vehicle but higher than that of Oseltamivir-treated cultures on day 4 (Figure 3C). The reduction in RANTES by the 1:5-diluted Pollival^®^ was similar to that of Oseltamivir on both day 2 and day 4, while a lower effect was detected at a 1:10 dilution (Figure 3D). Non-infected cultures treated with Pollival^®^ showed TEER values in the normal range (200–800 Ω-cm^2^) (Supplementary Figure S2C) and no cytotoxicity (<5%) was detected for 1:10-diluted Pollival^®^ on non-infected epithelia and only mild (6.5%) toxicity was detected with a 1:5-diluted nasal spray using LDH measurement on day 4 (Supplementary Figure S2D). 

## 4. Discussion

In this study, we evaluated the in vitro antiviral activity of azelastine-HCl against viruses from three unrelated viral families, namely Coronaviridae (SARS-CoV-2 and HCoV-229E), Pneumoviridae (previously called Paramyxoviridae, RSV) and Orthomyxoviridae (influenza A).

We previously demonstrated the efficacy of azelastine-HCl and a commercial nasal spray containing azelastine-HCl against major variants of SARS-CoV-2 in vitro [18]. The in vivo effectiveness of the azelastine-containing nasal spray was confirmed by a subsequent clinical trial [11]. Here, we show the activity of azelastine-HCl against the omicron BA.1 variant that emerged later during the SARS-CoV-2 pandemic in both co-administration and a therapeutic setting. Although the BA.1 variant has recently been replaced by BA.2 sub-lineages globally, we used BA.1 since it represents the most important features of omicron variants: a significant evasion from both vaccine and infection-induced antibodies [19,20] and an altered cell-entry pathway [21]. The unaltered activity of azelastine-HCl shown here against the BA.1 variant suggests that the extensive mutations in the spike protein are not relevant for the antiviral mechanism of action and a drastic change in efficacy is not expected against subsequent or newly emerging spike variants.

Furthermore, while pre-omicron variants used TMPRSS2 efficiently, the omicron BA.1 is associated with lower TMPRSS2 use [22]. This has been suggested to change the preference of BA.1 toward epithelial cells of the bronchi and the upper respiratory tract and correlate with lower virulence in animal models. This change in the cell entry mechanism could explain the lower effect of the serine protease inhibitor, nafamostat, against the BA.1 and future omicron variants [22]. In contrast to nafamostat, we observed the retained activity of azelastine-HCl against the BA.1 variant, indicating that this effect is independent of the utilization of TMPRSS2. 

The human seasonal coronavirus (HCoV) 229E belongs to the alpha coronaviruses, which bind to aminopeptidase N with its spike protein. Its genome is 65% homologous to that of SARS-CoV-2 and, consequently, prior exposure to seasonal HCoV viruses (including 229E) is a likely source of cross-reactive antibodies and the T cell response to SARS-CoV-2 in non-infected individuals. However, the protective role of these in a subsequent SARS-CoV-2 infection is debated [23,24,25]. Similarly, repeated vaccination against SARS-CoV-2 was shown not to induce a significant increase in specific antibodies against the HCoV-229E spike protein [26], suggesting that current vaccines against SARS-CoV-2 do not provide protection against seasonal coronavirus infections. Despite these antigenic differences, we have shown that azelastine-HCl, when administered simultaneously with the virus, reduced viral replication in vitro with an EC_50_ similar to that observed against SARS-CoV-2. The broad-spectrum activity of azelastine-HCl against viruses from the Coronaviridae family could be explained by targeting a shared virus component. Most approved antivirals are direct-acting antivirals (DAA) that mostly inhibit viral enzymes. A famous example is nirmatrelvir, the prodrug of the main protease (M^pro^) inhibitor developed against SARS-CoV-2. Recently, it was shown to effectively inhibit the replication of seasonal coronaviruses, including 229E, in various in vitro assays [27]. Azelastine-HCl was predicted in silico to inhibit the coronavirus protease M^pro^ and was later confirmed in vitro as well [28,29,30]; therefore, this direct antiviral mechanism could contribute to its activity against SARS-CoV-2 variants and HCoV-229E. However, the antiviral activity of azelastine-HCl has been confirmed beyond coronaviruses. As shown in this study, azelastine-HCl inhibited RSV infection both when applied together with virus inoculation and when used after viral infection (therapeutic setting). We also provide evidence that an azelastine-HCl-containing commercial nasal spray can inhibit the influenza A H1N1 infection of human nasal 3D tissue cultures, decreasing the viral count and the virus-induced cell damage (cilia damage) at early time points. As both RSV and influenza A lack the M^pro^ protease and rely on the use of host proteases, the mode-of-action (MoA) of azelastine-HCl against these viruses cannot be explained by the mechanism suggested above. 

Another strategy to achieve broad antiviral activity is to affect the host cell mechanisms used by multiple viruses. Such cell machinery includes protein folding and transport, cellular kinases, lipid metabolism, and cellular proteases [8]. This approach not only offers antiviral activity across different viral families, but the likelihood of emerging resistance is lower. Importantly, azelastine-HCl was previously shown to affect such host machinery. Firstly, Bamia et al. [31] showed that azelastine-HCl inhibited the protein-folding activity of the ribosome (PFAR) in *S. cerevisiae* and inhibited prion propagation; however, no direct inhibition of the heat shock proteins was shown, and a high concentration (over 50 µM) over a longer time was required for the effect. Secondly, azelastine-HCl, as a cationic amphiphilic drug (CAD), is expected to modulate the lipid-processing pathway at the same concentration range where we detected the antiviral effect of azelastine-HCl. We and others have previously discussed the relevance of this effect for SARS-CoV-2 [18,32]. CADs may affect the double-membrane vesicles used by positive single-strand RNA viruses for replication [33,34], which is a mechanism relevant for coronaviruses in general but not against RSV and influenza A. On the other hand, CADs may affect the endosomal–lysosomal change in the pH which is required for the conformational changes in hemagglutinin (HA) mediating influenza virus entry to the cytoplasm [35]. Unlike influenza viruses, the entry of RSV into the cytoplasm is pH insensitive [36]; therefore, this mechanism cannot explain the observed anti-RSV activity of azelastine-HCl. The interference of CADs with the lysosomal function also leads to the accumulation of cholesterol inside the late endosomal–lysosomal cell compartments [37], which is a documented host-cell protective mechanism inhibiting influenza A virus escape [38]. 

Besides the interference with viral enzymes or host machinery, azelastine may act by limiting the inflammation induced by viruses. Here, we demonstrated that in a human nasal tissue influenza infection model, azelastine significantly reduced the IL-8 and RANTES response of epithelial cells to H1N1. Whether this is due to a direct anti-inflammatory effect, or an indirect effect of the reduced viral count and a consequently lower cell disruption remains to be elucidated. Previously, azelastine-HCl was also shown to inhibit cytokine-induced ICAM-1 upregulation, which is a major receptor for Rhinoviruses and a potential receptor of RSV [39].

The mechanism of azelastine-HCl in exerting its antiviral activity is likely complex and may involve direct action on viruses, host cell pro-viral factors, and on the virus-induced host response (e.g., inflammation). 

Our study has the limitation that we relied on the use of in vitro models. To minimize this limitation, we applied a human nasal tissue 3D model to examine the effect of azelastine-HCl on influenza A. This model is highly translational, as it displays high trans-epithelial electrical resistance, cilia beating as well as mucus production, demonstrating the full functionality of the epithelial tissue. Additionally, there are clinical data that support our findings. We have shown in a phase 2 clinical study that the use of an azelastine-containing nasal spray reduced the viral load and shortened the viral carriage in the upper respiratory tract of SARS-CoV-2-infected individuals [11]. An earlier publication also showed the effect of the prophylactic use of an azelastine nasal spray to prevent upper respiratory tract infections in children [40]. Furthermore, a phase 2 study currently ongoing is using Pollival^®^ to prevent SARS-CoV-2 and further respiratory virus infections in adults (DRKS-ID: DRKS00031059, [41]) and may provide additional evidence in the near future.

## 5. Patents

CEBINA is the owner of patents derived from WO2021239943 related to the findings described in this paper.

## Figures and Tables

**Table 1:** Primers and probes used for virus detection.

Primer	Sequence	Reference
N protein (229E) fw	5′ TCTGCCAAGAGTCTTGCTCG 3′	[17]
N protein (229E) rev	5′ AGCATAGCAGCTGTTGACGG 3′	[17]
E gene (SARS-CoV-2) fw	5′ ACA GGT ACG TTA ATA GTT AAT AGC GT 3′	[18]
E gene (SARS-CoV-2) rev	5′ ATA TTG CAG CAG TAC GCA CAC A3′	[18]
E gene (SARS-CoV-2) probe	FAM-ACA CTA GCC ATC CTT ACT GCG CTT CG-BHQ1	[18]

## Data Availability

Raw data are available upon reasonable request from the corresponding author.

## Supplementary Materials

The following supporting information can be downloaded at: https://www.mdpi.com/article/10.3390/v15122300/s1, Table S1: Efficacy of azelastine-HCl against different viruses measured in vitro. Supplementary Figure S1: In vitro antiviral effect of azelastine-HCl against RSV administered at different time points relative to viral infection. Supplementary Figure S2: Tissue protection elicited by azelastine-HCl in an influenza A H1N1 infection model on human nasal 3D tissue.
