# Supplementary material for: Antiviral Potential of Azelastine against Major Respiratory Viruses
Source: Viruses. 2023 Nov 23;15(12):2300. doi: 10.3390/v15122300 (PMC10747764; doi:10.3390/v15122300)
Supplement: Supplementary file 1 [file viruses-15-02300-s001.zip › viruses-2708619-supplementary final.pdf]

Supplementary Table S1. Efficacy of azelastine-HCl against different viruses measured in vitro.

Virus	EC$_{50}$ (μM)		Reference
	Co-administration	Therapeutic	
**SARS-CoV-2 D614G**	3.7	4.0	[18]
**SARS-CoV-2 B.1.1.7**	2.8	4.3	[18]
**SARS-CoV-2 B.1.351**	5.5	6.5	[18]
**SARS-CoV-2 B.1.617.2**	5.4	4.6	[18]
**SARS-CoV-2 B.1.1.529**	2.8	3.8	This study
**HCoV-229**	3.4	n.d.	This study
**RSV Long**	1.038*-1.139**	n.d.	This study

EC50 was calculated with an inhibitor vs. response variable slope (four parameter). * using azelastine-HCl as a pure compound ** using azelastine-HCl containing the nasal spray Pollival®. n.d.—not determined.

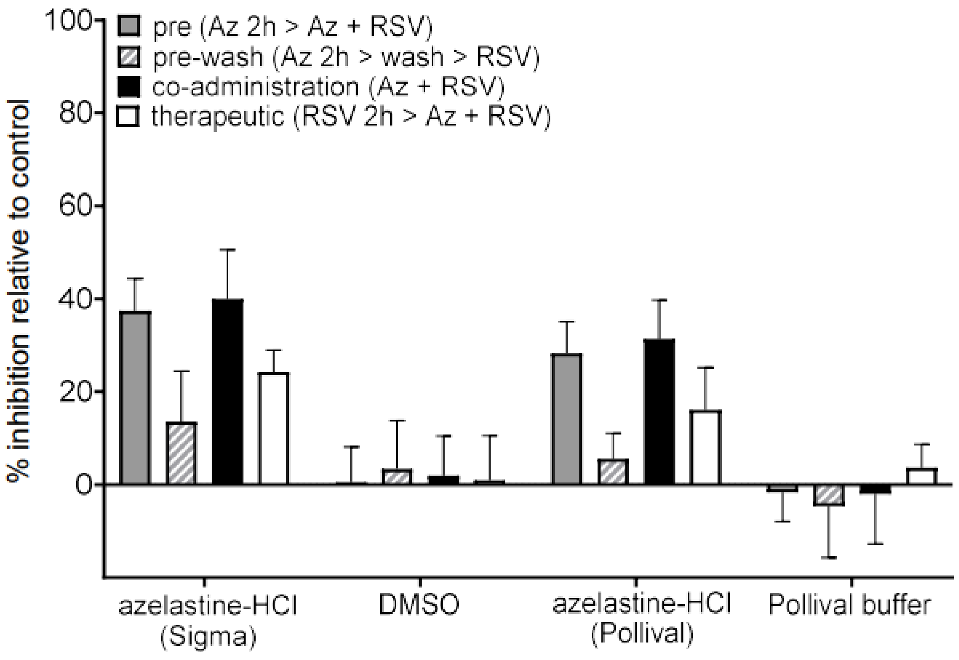

Supplementary Figure S1. *In vitro* antiviral effect of azelastine-HCl against RSV administered at different timepoints relative to viral infection. Pre indicates azelastine-HCl treatment 2 hours prior infection and during infection (grey bar), pre-wash means administration for 2 hours was followed by wash prior infection (bars with stripes), co-administration represents treatment at the time of infection (black bars) and therapeutic administration indicates treatments 2 hours post-infection (white bars). Graphs show the mean with 95% CI from 5-10 independent experiments.

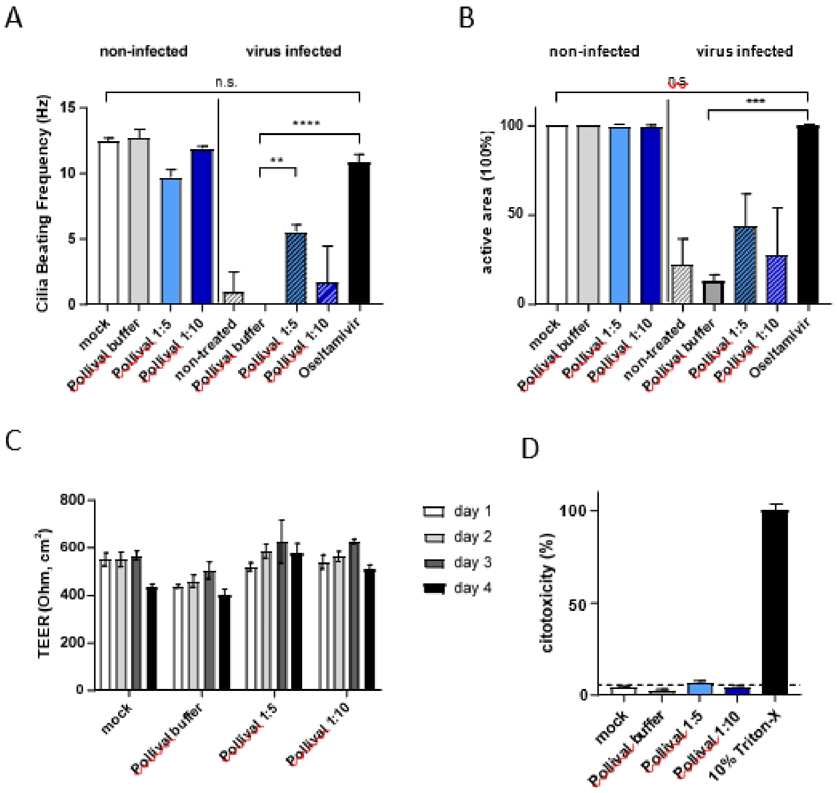

Supplementary Figure S2. Tissue protection elicited by azelastine-HCl in an influenza H1N1 infection model on human nasal 3D tissue. (A) Cilia Beating Frequency (CBF) measured 96h p.i. (B) Area with active cilia expressed as percentage of total tissue area assessed 96h p.i.. (C), (D) Potential tissue damage caused by the azelastine-HCl nasal spray evaluated on tissues non-infected by H1N1 virus but treated repeatedly with 1:5 or 1:10 diluted Pollival; (C) tissue integrity measured by TEER on each experimental day, (D) citotoxicity measured based on LDH level in tissue media 96h after experiment start and compared to non-treated tissues. Citotoxicity is considered significant if above 5%. Difference between groups was statistically analysed with one-way ANOVA (multiple comparison) using GraphPad Prism and considered significant if $p < 0.05$. * * $p < 0.01$; * * * $p < 0.001$; * * * * $p < 0.0001$; n.s. Indicated non-significant difference. Graphs show the mean ± SD of results from 3 tissue samples.
